# A Novel Variant of the *CHD8* Gene in a Patient with Autism Spectrum Disorder

**DOI:** 10.3390/genes17060599

**Published:** 2026-05-23

**Authors:** Elena Falcone, Alessia Bauleo, Laura De Stefano, Rossella Brando, Sabrina Maietta, Elisabetta Tabolacci, Alberto Montesanto, Vincenza Pace, Rosalbina Apa, Domenica Puntorieri, Luca Cento, Giada Cuconato, Maria Grazia Muoio, Maurizio Genuardi

**Affiliations:** 1 BIOGENET, Laboratorio di Genetica Medica e Forense Cosenza, 87100 Cosenza, Italy; bauleo@biogenet.it (A.B.); lauradestefano@virgilio.it (L.D.S.); rossellabrando@yahoo.it (R.B.); vincenza_pace@yahoo.it (V.P.); rosa.apa@hotmail.it (R.A.); giada.cuconato01@universitadipavia.it (G.C.); muoiomg@gmail.com (M.G.M.); 2 Dipartimento di Scienze della Vita e Sanità Pubblica, Università Cattolica del Sacro Cuore, 00168 Roma, Italy; sabrina.maietta01@icatt.it (S.M.); elisabetta.tabolacci@unicatt.it (E.T.); maurizio.genuardi@unicatt.it (M.G.); 3 UOC Genetica Medica, Fondazione Policlinico Universitario A. Gemelli IRCCS, 00168 Roma, Italy; 4 Dipartimento di Biologia, Ecologia e Scienze della Terra, Università della Calabria, 87036 Rende, Italy; alberto.montesanto@unical.it; 5 Dipartimento Materno Infantile Neuropsichiatria Infanzia e Adolescenza Rossano—Cariati, Azienda Sanitaria Provinciale di Cosenza, 87064 Cosenza, Italy; d.puntorieri@aspcs.gov.it; 6 Associazione Equilibri Pedagogici, Studio Pedagogico Interdisciplinare, 89129 Reggio Calabria, Italy; luca.cento@gmail.com; 7 Dipartimento di Medicina Molecolare, Università degli studi di Pavia, 27100 Pavia, Italy

**Keywords:** CHD8, ASD, macrocephaly, exome, variant

## Abstract

**Background/Objectives**: Autism spectrum disorder (ASD) is a neurodevelopmental disease with both clinical and genetic heterogeneity. Several loss-of-function variants in the chromodomain helicase DNA-binding protein 8 (*CHD8*) gene have been identified in individuals with ASD and/or developmental delay/intellectual disability. These are associated with specific clinical manifestations, including overgrowth, macrocephaly, sleep disturbance, and gastrointestinal problems. **Methods**: We performed clinical exome sequencing in a female patient with ASD and macrocephaly. RNA analysis from peripheral blood was carried out to investigate the functional effect of the identified variants. **Results**: We identified a novel maternally inherited *CHD8* variant (c.5390+2T>C). Transcript analysis demonstrated that this variant disrupts the canonical splice donor in intron 30, causing splicing anomalies in the CHD7-binding domain of the CHD8 protein, resulting in a truncated inactive protein. **Conclusions**: In conclusion, this study identified a novel splice-site variant in the *CHD8* gene with experimentally confirmed pathogenic effects on RNA splicing, expanding the mutational spectrum of CHD8-related neurodevelopmental disorders. The considerable intrafamilial phenotypic variability associated with CHD8 haploinsufficiency supports the presence of reduced penetrance and highlights the influence of modifying factors on the clinical expression of CHD8-related disorders.

## 1. Introduction

Autism Spectrum Disorder (ASD) is a common neurodevelopmental disorder characterized by impairments in social communication and the presence of restricted or repetitive behaviors or interests, with a strong but complex genetic component [1,2]. ASD is highly heritable, influenced by a combination of rare de novo and inherited variants, including chromosomal abnormalities, copy number variants (CNVs), and single-nucleotide variants (SNVs), as well as common variants in at least several hundred genes, clustered within genetic loci associated with ASD [3,4,5]. Epigenetic regulation represents an additional critical layer influencing gene expression and contributing to the multifactorial etiology of ASD onset. Up to now, more than 100 high risk ASD genes have been identified and shown to converge on shared molecular pathways, regulating neuronal differentiation, circuit formation, and activity-dependent plasticity [1,2]. Loss-of-function variants of the *CHD8* (OMIM #610528) gene are associated with ASD [6,7]. A wide spectrum of *CHD8* pathogenetic variants (PVs) has been observed in individuals with ASD, including chromosomal microdeletions [6,7], balanced chromosomal abnormalities [8], and *de novo* loss-of-function (LoF) variants [3,4,6,7,9,10,11,12,13,14]. Common phenotypic manifestations, namely overgrowth, distinctive facial features, macrocephaly, sleep disturbance, and gastrointestinal problems, suggestive of a syndromic form of ASD, have been consistently recognized in carriers of *CHD8* PVs [6,12].

The *CHD8* gene, located on chromosome 14q11.2, encodes a SNF2-like member of the chromodomain helicase DNA-binding protein family, which plays a critical role in chromatin remodeling and transcriptional regulation. The CHD8 protein consists of 2581 amino acid residues and contains five conserved functional domains (CHROMO, SNF2_N, HELIC, CHD7_BD, and BRK [15]), including core domains to remodel chromatin structure (SNF2_N and HELIC) with two tandem chromodomains (CHROMO) located in the N-terminal region, responsible for binding to methylated histone H3 lysine 4 (H3K4me), alongside BRK (Brahma and Kismet) and CHD7_BD domains, which allows protein–protein interaction. The protein acts as a DNA helicase that remodels chromatin structure and in particular, interacts and regulates the co-expression of other proteins encoded by ASD risk genes during brain development [16,17,18,19]. Inactivated *CHD8* variants have been reported both as germline alterations in ASD and as somatic events in cancer [15,20,21,22]. In particular, variant distribution across protein domains seems to cluster similarly on CHROMO and HELIC domains for *CHD8* LOFs variants in ASD vs. cancer groups, but a relative enrichment of *CHD8* missense variants in the SNF2_N domain, and a decrease in the CHD7_BD domain, was only seen in the cancer group. Nevertheless, LOFs variants in ASD are present at significantly higher proportions [5].

To date, more than 330 *CHD8* variants have been identified in ASD patients (https://gene.sfari.org/, SFARI gene score 1S last access 14 March 2026).

Although several *CHD8* splice-site variants have previously been reported in individuals with ASD [2,15], functional characterization at the RNA level remains limited for many of these alterations, and the molecular consequences of inherited splice-disrupting variants are still incompletely understood. In particular, most published studies have focused on *de novo* loss-of-function variants, whereas maternally inherited canonical splice-site variants with experimentally validated effects on transcript processing are rarely documented. Therefore, detailed molecular characterization of novel splice-altering *CHD8* variants is important to refine genotype–phenotype correlations and improve interpretation of non-coding pathogenic mechanisms in ASD.

Here, we describe an ASD patient carrying a novel maternally inherited *CHD8* single nucleotide variant affecting a canonical splice-site consensus sequence. RNA analysis demonstrated that this variant disrupts normal splicing, resulting in the production of a nonfunctional allele.

## 2. Materials and Methods

### 2.1. Patients and Samples

Written informed consent was obtained from both parents of the patient to perform molecular analyses as part of routine clinical investigations, and to share anonymized clinical information and clinical photographs.

### 2.2. Molecular Analyses

Genomic DNA was extracted from peripheral blood leukocytes of the proband and her parents using the Qiagen DNAeasy spin-column kits on a semi-automatic QIAcube instrument (Qiagen Hilden, Hilden, Germany). DNA was quantified using the QuBit™1X dsDNA HS Assay Kit using a QuBit Fluorimeter (Thermo Fisher, Waltham, MA, USA).

Libraries preparation was performed using the Clinical Exome Solution (CES) kit (Sophia Genetics SA, Saint-Sulpice, Switzerland), which targets 4490 known disease-associated genes, according to the manufacturer’s instructions. Library quality and quantity were assessed using D1000 and High Sensitivity D1000 Reagents Kits on a TapeStation 4150 system (Agilent Technologies, Santa Clara, CA, USA). Normalized libraries were sequenced on an Illumina MiSeq platform using a MiSeq Reagent kit v3 (2 × 300 × cycles) (San Diego, CA, USA). Base calling and quality scoring were performed using the manufacturer’s real-time analysis software, followed by the generation of FASTQ sequence files (mapped reads: 98.88%, target regions 100×: 75.02%). Reads were mapped to the GRCh37/hg19 genome.

A virtual panel of 187 genes implicated in ASD was applied for the secondary bioinformatic analysis. Variant annotation, filtering, and interpretation were performed using the Sophia DDM™ platform (Sophia Genetics SA) according to the American College of Medical Genetics and Genomics (ACMG) guidelines [23] and ACGS best practice guidelines for variant classification in rare disease [24].

The variant identified in the proband was confirmed by Sanger sequencing. Genomic DNA from the proband and her parents was amplified using the following primers: forward 5′-AGGCAGAAAAGATGGTCCCT-3′ and reverse 5′-AGATAGAGGCTGCAGAACGT-3′, targeting the CHD8 splice-site variant (NM_001170629.1: c.5390+2T>C;). Sequencing reactions were performed using the BigDye™ Terminator v.3.1 Cycle Sequencing Kit (Applied Biosystems, Life Technologies, Waltham, MA, USA), followed by capillary electrophoresis on a SeqStudio Genetic Analyzer (Applied Biosystems, Life Technologies, USA). Sequence analysis was carried out with Sequencher v5.4.6 (Gene Codes Corporation, Ann Arbor, MI, USA).

Total RNA was extracted from short-term phytohemagglutinin (PHA)-stimulated leucocyte cultures established from peripheral blood samples of the proband and from two unrelated control individuals undergoing routine preconception carrier screening, with no reported history of neurodevelopmental disorders. Cells were cultured in RPMI medium for 96 h in two subcultures: untreated and treated with puromycin (Merck, Darmstadt, Germany) at a final concentration of 0.2 mg/mL for the last 4 h of incubation to inhibit nonsense-mediated mRNA decay (NMD). At the end of the treatment, total RNA was extracted using TRIzol protocol (Thermo Scientific, Waltham, MA, USA). A total of 500 ng of total RNA were reverse transcribed with ImProm-II Reverse Transcription System kit (Promega, Madison, WI, USA).

cDNA was amplified with coding primers that encompassed intron 30 of the *CHD8* gene (NM_001170629.1). The following primer pairs were used: forward primer 5′-AGATAGAGGCTGCAGAACGTG-3′ (exon 30), and reverse primer 5′-AAAGACAGATGAAAGCCTTACCA-3′ (exon 31); an additional forward primer located on exon 29 was also used together with the reverse primer on exon 31 (5′-TCCCTTGATGATGATGGATG-3′). PCR products obtained from untreated and puromycin-treated samples were analyzed on a 4% agarose gel. Amplicons derived from the proband were extracted and purified with StrataPrep DNA Gel Extraction Kit (code 400766, Agilent Technologies, Santa Clara, CA, USA) and subsequently cloned using the StrataClone PCR Cloning Kit (code 240205, Agilent Technologies, Santa Clara, CA, USA). White colonies were selected and sequenced using T3 and T7 plasmid-specific primers.

## 3. Results

### 3.1. Clinical Description

The patient was an Italian female who was first evaluated at 11 years of age with a diagnosis of ASD according to criteria from the *Diagnostic and Statistical Manual of Mental Disorders, Fifth Edition* [25]. A comprehensive clinical evaluation including a physical and dysmorphological examination was performed, and detailed information regarding pregnancy, medical, family, and social histories was collected.

**Figure 1:**
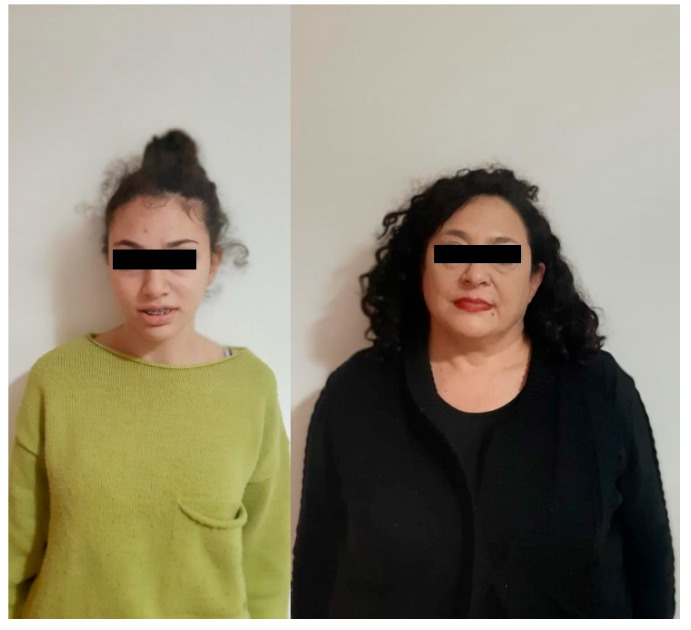
Pictures of the proband and her mother. On the left, the proband at 15 years of age showing dysmorphic features, including hypertelorism, large ears, a prominent forehead, a pronounced supraorbital ridge, and a pointed chin. On the right, the patient’s unaffected healthy mother, carrier of the same CHD8 variant.

At the most recent physical examination she was 15 years old and showed macrocephaly (head circumference 57 cm), tall stature (height 170 cm) and weight in the 78th percentile. Facial features included hypertelorism, large ears, a prominent forehead, a pronounced supraorbital ridge, and a pointed chin (Figure 1). Neuropsychological assessment was performed using the *Wechsler Intelligence Scale for Children, Fourth Edition* (WISC-IV). This showed a non-impaired cognitive function (Verbal Comprehension Index VCI = 94, Perceptual Reasoning Index PRI = 65, Working Memory Index WMI = 109, Processing Speed Index PSI = 71, and Full Scale IQ = 79). Anomalies in executive functioning, including complex motor tics, emotional lability, mood disturbances, anxiety, impairments in nonverbal communicative behaviors used for social interaction, and deficits in praxic motor organization and complex praxis, were also noticed. Physical examination of the mother also showed macrocephaly (head circumference 59 cm) and tall stature (height 167 cm). Psychological assessments were performed using standardized self-report questionnaires. Results from the Symptom Checklist-90-Revised (SCL-90-R) indicated clinically significant somatization (SOM = 60), whereas no evidence of increased anxiety sensitivity was observed on the Anxiety Sensitivity Index-3 (ASI-3). Alexithymia was excluded based on the Toronto Alexithymia Scale-20 (TAS-20).

### 3.2. Genetic Analyses

Clinical exome sequencing identified a heterozygous *CHD8* variant, NM_001170629.1:c.5390+2T>C, in intron 30, predicted to result in a frameshift and premature termination p.(Trp1758Glnfs*9). The variant is located at the canonical donor splice site of intron 30, within the region encoding the CHD7-binding domain of the CHD8 protein. The presence of the variant was confirmed by Sanger sequencing, and segregation analysis showed that it was inherited from the proband’s mother.

**Figure 2:**
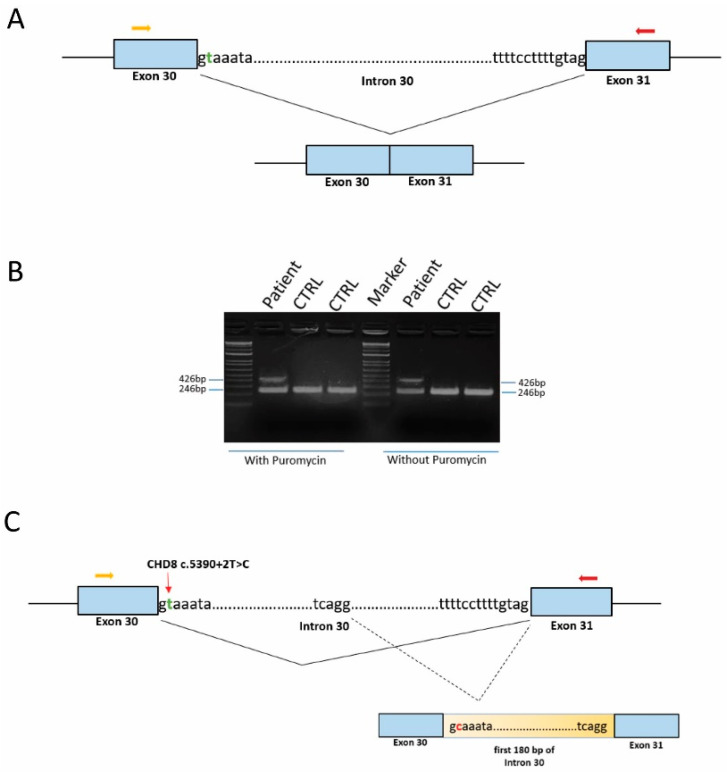
Effects on transcription of the *CHD8* c.5390+2T>C variant. (**A**) Schematic representation of the normal splicing pattern in the region of interest, between exon 30 and 31. Arrows represent the primers used to amplify and sequence the region; upper and lower-case letters indicate exonic and intronic nucleotides, respectively. (**B**) After cDNA amplification, a single 246 bp band is visible in two independent controls, while two bands, one corresponding to the 246 bp wild-type product and another to a longer 426 bp band, were detected in the proband’s mRNA. The two bands found in the patient were extracted from agarose gel, cloned, and sequenced. (**C**) Schematic representation of the aberrant splicing caused by the *CHD8* heterozygous c.5390+2T>C variant. The lower 246 bp band corresponds to the normal transcript (canonical splicing exons 30–31) (on the left), while the upper band of 426 bp revealed the presence of 180 nucleotides of intron 30 (on the right). The variant abolished the canonical donor splice site, leading to the activation of a downstream cryptic splice site within intron 30. The presence of transcripts other than those sequenced was excluded through the amplification and sequencing of the patient and controls’ cDNA using a different primer pair (between exons 29 and 31).

The c.5390+2T>C variant was not reported in public reference databases, including ClinVar and HGMD, and was absent from the Genome Aggregation Database (gnomAD v4.1.1 last access 14 May 2026). The variant is located in the consensus sequence of the donor splicing site involved in the removal of intron 30 (Figure 2A), leading to a disruption of reading frame, and removing a region critical to protein function (CHD7-binding domain) [26,27]. The DECIPHER database was used to predict NMD escape of the splicing variant (v11.38). In silico analysis with Human Splicing Finder software (http://www.umd.be/HSF/HSF.shtml, Version 3.1, last access 14 March 2026) predicted a severe disruption of the donor splice site of intron 30, strongly supporting a pathogenic effect on pre-mRNA splicing. According to the ACMG and ACGS criteria, the c.5390+2T>C variant can be classified as pathogenic (criteria applied: PVS1_strength (RNA) and PM2) [23,28].

### 3.3. RNA Analysis

To further evaluate the functional impact of the c.5390+2T>C variant, which was predicted by in silico analysis to affect splicing, RNA analysis was performed. cDNA amplification using primers spanning exons 30 and 31 revealed two PCR products of 426 and 246 bp in the proband, detected both in untreated and puromycin-treated conditions, suggesting the presence of two distinct splicing isoforms. In contrast, only a single PCR product, corresponding to the canonical transcript (246 bp), was observed in all control samples (Figure 2B). Sequencing of cloned PCR products from the proband demonstrated partial retention of the first 180 nucleotides of intron 30 in the 426 bp fragment, whereas the 246 bp fragment corresponded to the canonical splicing pattern (Figure 2C). Comparable results were obtained in the absence of puromycin treatment, suggesting that the aberrant transcript may be at least partially resistant to nonsense-mediated mRNA decay and/or not efficiently targeted by this pathway, although we cannot exclude a contribution of NMD based on the available qualitative data. These findings indicate that the c.5390+2T>C variant abolishes the canonical donor splice site and leads to the activation of a cryptic downstream donor site within intron 30. As expected, analysis of control samples confirmed the exclusive presence of the correctly spliced transcript (246 bp. The aberrant transcript is predicted to introduce a frameshift, resulting in a premature termination codon, and to encode a truncated, nonfunctional protein (p.Trp1758Glnfs*9).

These functional data indicate that the c.5390+2T>C variant is functionally deleterious, thereby supporting its classification as a pathogenic variant [23,28].

## 4. Discussion

ASD is a genetically heterogeneous condition caused by rare and common variants across hundreds of genes, with many high-confidence risk genes converging on key neurodevelopmental pathways, including synaptic function and chromatin regulation [29,30].

Genomic studies based on exome sequencing and targeted analysis have identified approximately 100 high-confidence genes with high penetrance for ASD, many of which are enriched for de novo loss-of-function (LoF) variants, including *CHD8* [15]. *CHD8* has emerged as one of the strongest ASD risk genes based on the excess of *de novo* disruptive variants in affected individuals, and their marked depletion in the general population [31]. The CHD8 protein is an ATP-dependent chromatin remodeling protein that plays a crucial role during brain development [32]. Cotney and colleagues demonstrated that in humans, CHD8 directly binds and regulates the co-expression of multiple ASD risk genes during human brain development, some of which are also involved in chromatin regulation [19]. Consequently, reduced CHD8 function can lead to the dysregulation of directly bound ASD risk genes (direct effect), thereby altering neurodevelopmental trajectories. Additional mechanisms have also been suggested. Sugathan et al. showed that the loss of CHD8 also affects the expression of ASD risk genes without a CHD8 binding site, suggesting the existence of indirect regulatory mechanisms [30]. Several hypotheses have been proposed to explain how CHD8 may be able to influence gene activation or transcription factor activity without directly binding to DNA. Even if the exact mechanisms are not completely understood, CHD8 is thought to influence gene expression through interactions with additional chromatin modifiers or transcriptional co-regulators [33].

Beyond behavioral features consistent with ASD, individuals carrying pathogenic *CHD8* variants share a constellation of phenotypic traits suggestive of a recognizable ASD subtype [6]. In fact, patients with *CHD8* variants also show other co-existing phenotypic findings including macrocephaly, rapid postnatal growth, distinctive facial features (prominent forehead, wide-set eyes, pointed chin), and gastrointestinal problems [6]. These clinical observations are supported by animal models, in which CHD8 haploinsufficiency in mice lead to macrocephaly, craniofacial abnormalities, learning and memory defects, and autism-like phenotype [34,35,36], while CHD8 disruption in zebrafish leads to an increased head size and gastrointestinal motility problems [30].

In the present study, we identified a novel LoF variant in *CHD8*, NM_001170629.1:c.5390+2T>C, affecting the canonical donor splice site of intron 30, in a patient diagnosed with ASD. In silico analysis predicted a severe disruption of normal splicing, which was confirmed by RNA studies. Functional assays demonstrated abolition of the canonical donor splice site and activation of a cryptic downstream splice site within intron 30, resulting in partial intron retention and generation of an aberrant transcript. This transcript introduces a frameshift and a premature termination codon, leading to a truncated, non-functional protein. These functional data provide strong evidence for a deleterious effect of the c.5390+2T>C variant and support its classification as pathogenic, according to ACMG/ACGS guidelines, based on its absence from population databases, predicted loss-of-function effect in a gene with established haploinsufficiency, and experimental demonstration of abnormal splicing.

Clinically, the proband exhibited ASD with borderline cognitive functioning, and prominent executive dysfunction, including attention deficit, complex motor tics, emotional lability, mood and anxiety symptoms, impaired nonverbal social communication, and deficits in motor planning and praxis. Many of these clinical features overlap with those previously reported for patients with *CHD8* variants. However, the absence of intellectual disability, language delay, and gastrointestinal or sleep disturbances in our patient highlights the phenotypic variability associated with CHD8 haploinsufficiency. The variant was inherited from the healthy mother, who did not meet criteria for ASD and displayed a mild phenotype with macrocephaly and minor psychological features on standardized testing.

This intrafamilial variability indicates the incomplete penetrance and variable expressivity of the c.5390+2T>C variant. Despite the high penetrance of heterozygous *CHD8* variants with respect to other ASD-linked genes, almost 20% of individuals do not appear to have clear neurodevelopmental or psychiatric symptoms [2]. Recent studies have begun to shed some light on the potential modifiers of *CHD8* haploinsufficient mutations. Both cellular experiments on CHD8 patient-derived iPSC-derived neurons and on in vivo mouse models provided direct experimental proof that genetic background modifies neurodevelopmental phenotypes [37,38]. Furthermore, it is intriguing to note that *CHD8* variants appear to disproportionately affect males. An explanation might be that females with a *CHD8* variant are less severely affected and therefore less frequently diagnosed and genetically tested [2,10]. Moreover, mice experiments demonstrate a sex-specific effect on transcriptional regulation and phenotype, with male mice being more affected [39]. In agreement with those studies, we also observed a lower phenotypic expression in this family.

A limitation of the study is that array-based comparative genomic hybridization (aCGH) was not performed on the proband and the mother. We could not exclude the presence of copy number variant (CNV) that may further contribute to patient phenotype severity.

## 5. Conclusions

This study provides functional validation of a novel splice-site variant in the *CHD8* gene and further expands the mutational and phenotypic spectrum associated with CHD8-related ASD. Our results highlight the importance of RNA-based assays for accurate variant interpretation and emphasize the substantial clinical heterogeneity and variable expressivity associated with *CHD8* PVs.

## Data Availability

The data that support the findings of this study are available on request from the corresponding author.
